# Multisystem Embolic Events From Prosthetic Mitral Valve Endocarditis: A Case Report

**DOI:** 10.7759/cureus.92174

**Published:** 2025-09-12

**Authors:** Mehdi Berrajaa, Ihsene Cherradi El Fadili, Hicham Eddakch, Wassim Beladel, Khalil Abderrahmane Elbaz, Mohamed El Minaoui

**Affiliations:** 1 Cardiology Department, Souss-Massa University Hospital, Faculty of Medicine and Pharmacy, Ibn Zohr University, Agadir, MAR

**Keywords:** complicated infective endocarditis, endocarditis, prosthetic mitral valve, prosthetic valve endocarditis, systemic emboli

## Abstract

Prosthetic valve endocarditis (PVE) is a severe form of infective endocarditis, associated with high morbidity, mortality, and frequent embolic complications. Embolic events are among its most severe complications, potentially involving major organs such as the brain, spleen, kidneys, and lungs. We report herein the case of a 58-year-old woman with a mechanical mitral valve who presented with fever, constitutional symptoms, and subsequent acute right-sided hemiparesis and dysarthria. Transthoracic and transesophageal echocardiography revealed a large, mobile vegetation on the prosthetic mitral valve. Imaging confirmed multiple systemic emboli involving the spleen, right kidney, and superior mesenteric artery. Blood cultures, including fungal studies, were repeatedly negative, but the diagnosis of PVE was established using the modified Duke criteria. Our patient received empiric intravenous antibiotherapy, resulting in clinical improvement and a reduction in inflammatory markers. Despite favorable medical response, the presence of a large vegetation and multiple embolic events warranted urgent surgical reassessment. The rarity of this case lies in the occurrence of multiple embolic sites during the same episode of endocarditis. It underscores the aggressive nature of PVE, its propensity for multisystemic embolic events, and the critical importance of early diagnosis to optimize patient outcomes.

## Introduction

Infective endocarditis (IE) is a severe infection of the endocardial surface, most often involving the heart valves [1], with a complex pathogenesis [2]. Mechanical prosthetic valves are associated to a higher thrombotic risk, requiring lifelong anticoagulation [3]. Endocarditis involving a prosthetic cardiac valve is one of the most severe forms of IE [2], with mortality rates of 22% to 40% [4]. Prosthetic valve endocarditis (PVE) occurs in up to 2% of prosthetic valve recipients per year [5]. Embolic events occur in about 25% of PVE cases at diagnosis [2], most often affecting the brain, spleen [1], kidneys, and lungs. Multiple risk factors have been reported, including large (>10 mm) and mobile vegetations, mitral or right-sided location and elevated CRP [2]. We report here the case of a 58-year-old woman with a mechanical mitral valve who developed culture-negative PVE with a large, mobile vegetation and multiple systemic emboli. This case underscores the importance of early recognition and prompt management of PVE, particularly when complicated by systemic embolization. Awareness of current guideline recommendations is essential to optimize patient outcomes in this high-risk setting.

## Case presentation

We report the case of a 58-year-old woman with a history of mechanical mitral valve replacement in 2015 for severe mitral regurgitation and recently diagnosed type 2 diabetes. Twenty-five days prior to admission, she developed a dry cough, intermittent fever, and progressive constitutional symptoms, including fatigue, anorexia, and weight loss. Fifteen days later, while the fever persisted, she experienced the sudden onset of right-sided hemiparesis and dysarthria, which prompted her hospitalization at a regional medical center.

On admission to our cardiology department, the patient was alert, afebrile, and hemodynamically stable (heart rate: 78 bpm; blood pressure: 125/75 mmHg). Respiratory status was also stable (16 breaths per minute), with no clinical signs of heart failure. Neurological examination revealed partial motor weakness of the right upper limb. Cardiac auscultation demonstrated well-audible prosthetic valve clicks without murmurs. Electrocardiography showed atrial fibrillation with a ventricular rate of 78 bpm. Transthoracic echocardiography (TTE) subsequently revealed a vegetation attached to the prosthetic mitral valve.

**Figure 1:**
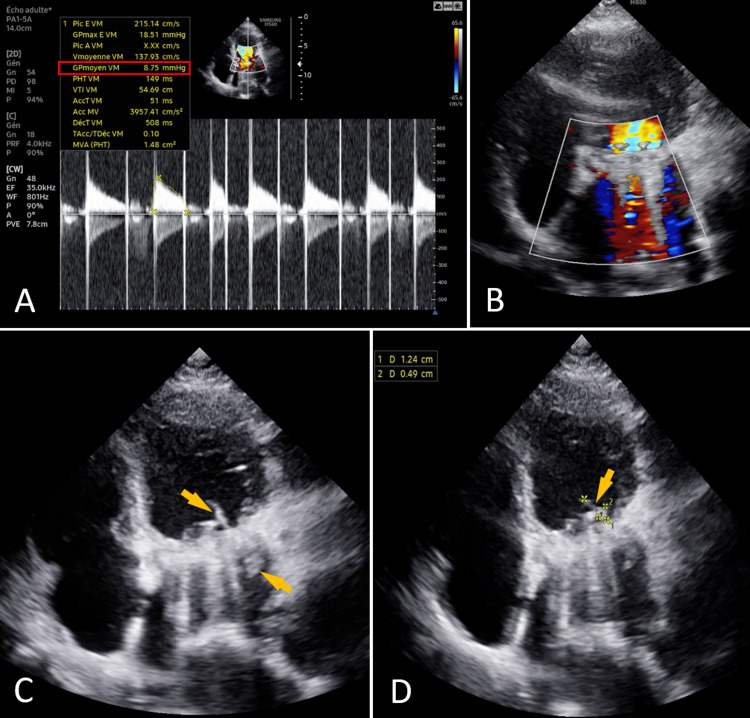
A: Elevated trans-prosthetic gradients (8.75 mmHg) on continuous Doppler, suggesting prosthetic dysfunction. B: TTE apical 4-chamber view suggesting obstruction of the mechanical mitral prosthesis on color Doppler. C and D: TTE apical four-chamber view: Vegetations on the bileaflet mechanical prosthesis in the mitral position (Arrows). TTE: Transthoracic Echocardiography

Initial TTE revealed a preserved left ventricular ejection fraction of 61%, without ventricular dilation or hypertrophy. The mitral prosthesis appeared dysfunctional, with a mean transvalvular gradient of 8.75 mmHg and an effective orifice area of 1.48 cm² (Figure 1A). Spontaneous contrast was observed in the left atrial appendage, suggesting impaired atrial flow (Figure 1B). A mobile echo-dense mass consistent with vegetation was visualized on both the ventricular and atrial sides of the prosthesis, measuring 12.4×0.49 mm, raising strong suspicion for infective endocarditis (Figure 1C, 1D).

**Figure 2:**
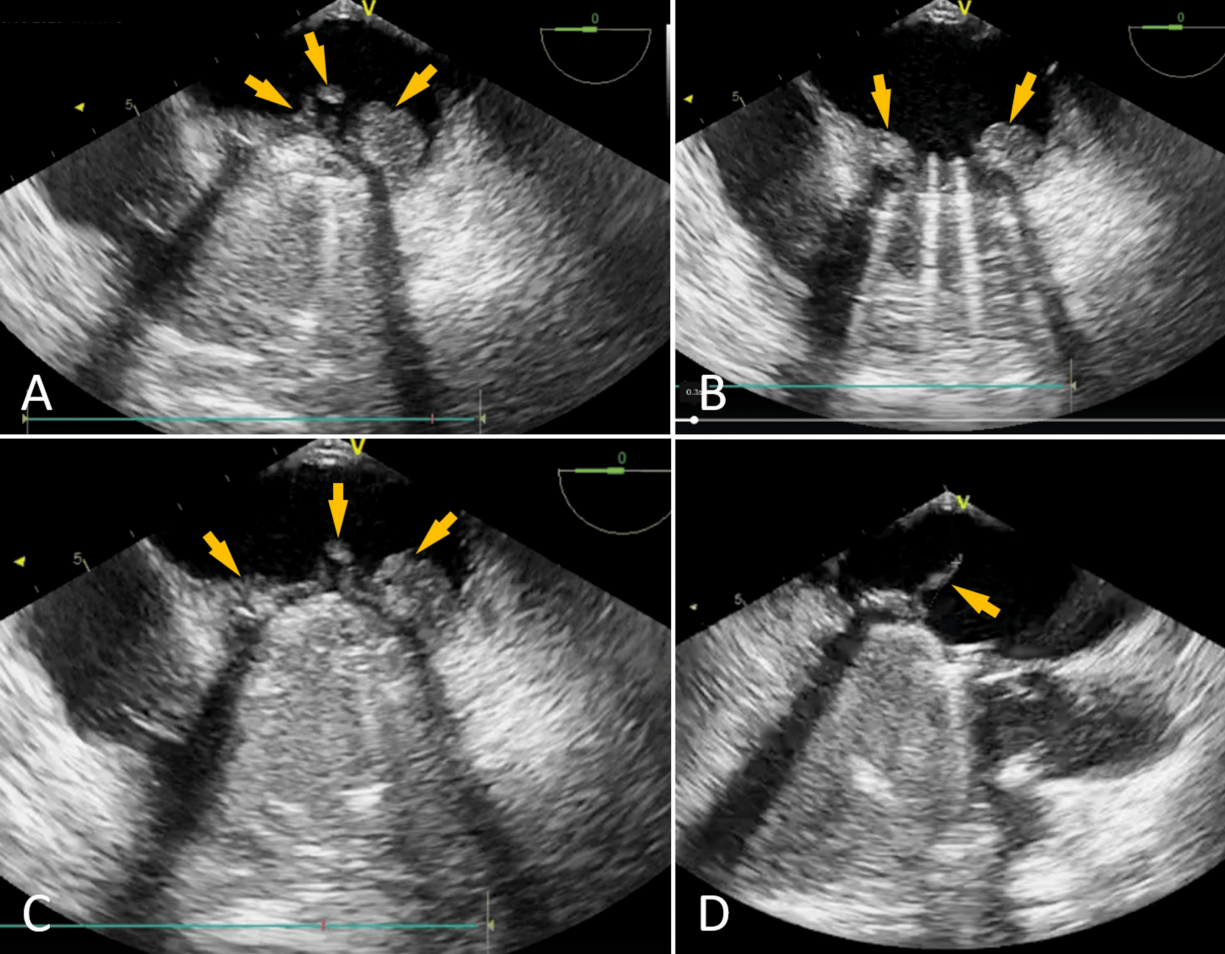
A, B, C: TEE four-chamber view, mid-esophageal: Large vegetation on the atrial side of the mechanical mitral prosthesis (Arrows). D: TEE cardiac long-axis view, mid-esophageal: Serpentine vegetation measuring 16 mm (Arrow) on the atrial side of the mechanical mitral prosthesis. TEE: Transesophageal Echocardiography

Transesophageal echocardiography (TEE) confirmed a large, polylobulated, highly mobile vegetation on the atrial side of the prosthesis, measuring 14×26 mm, with two elongated mobile components, the largest measuring 16 mm, features associated with high embolic risk (Figure 2A-2D).

**Figure 3:**
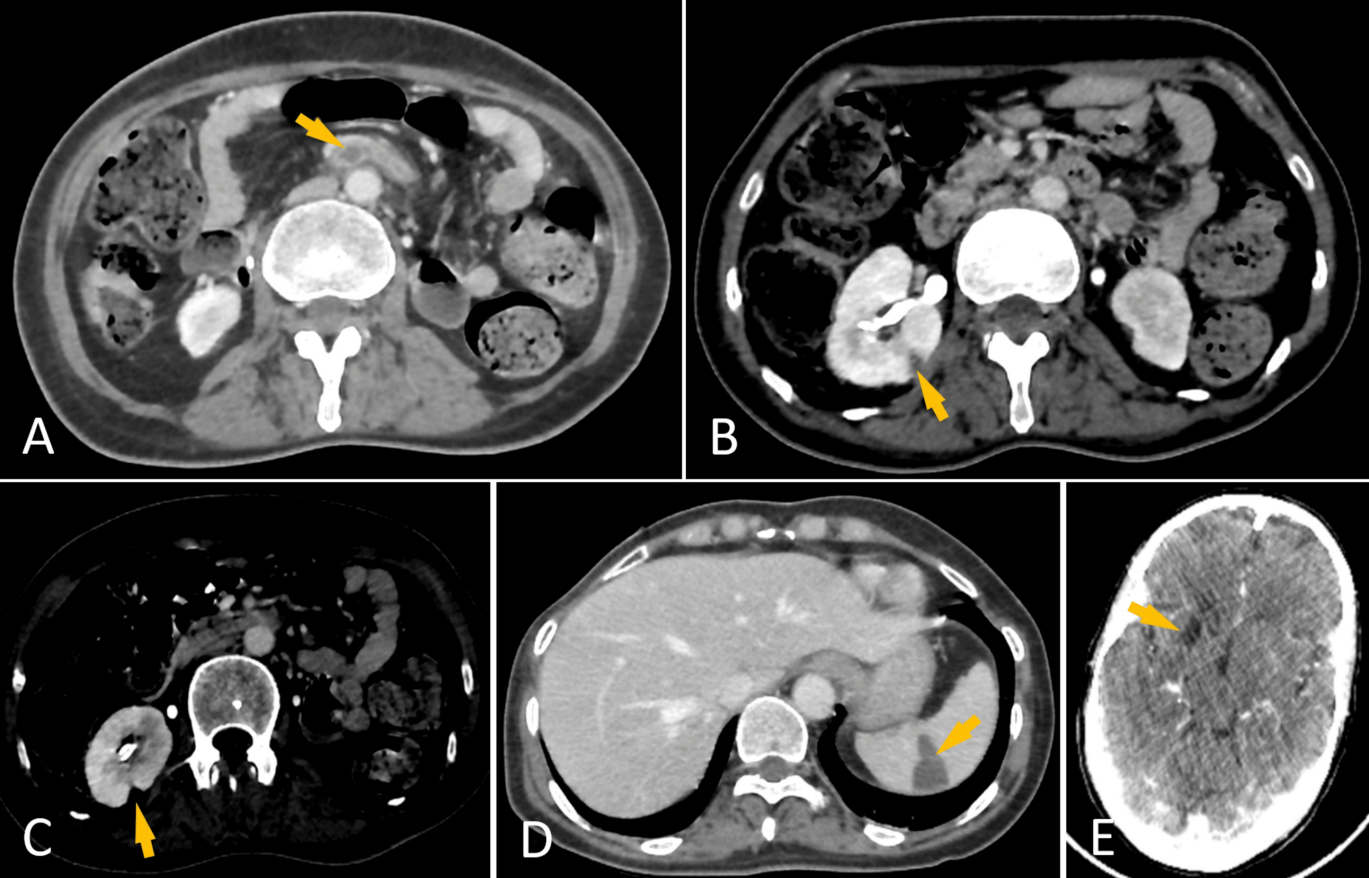
A: Axial abdominal CT scan: Luminal defect of the superior mesenteric artery, consistent with thromboembolism (Arrow). B: Axial abdominal CT scan: Wedge-shaped hypodense area of the right kidney, indicating renal infarction (Arrow). C: Axial abdominal CT scan: Wedge-shaped hypodense area of the right kidney, indicating renal infarction (Arrow). D: Axial abdominal CT scan: Hypodense, wedge-shaped area in the spleen, consistent with splenic infarction (Arrow). E: Axial brain CT scan: Right caudo-lenticulo-capsular ischemic lesion (Arrow).

Thoracoabdominopelvic CT revealed multiple systemic emboli involving the superior mesenteric artery (Figure 3A), right kidney (Figure 3B, 3C), and spleen (Figure 3D), consistent with septic embolization. Brain MRI was contraindicated due to a non-MRI-compatible prosthesis. Cranial CT demonstrated a subacute ischemic lesion, most likely representing sequelae of a prior embolic event (Figure 3E). Follow-up TTE after the embolic events demonstrated a reduction in the size of residual intracardiac masses. The prosthesis was well seated and functional, with a mean gradient reduced to 3 mmHg, suggesting embolization of the previously observed vegetation.

Despite multiple negative blood cultures, including fungal studies, the diagnosis of infective endocarditis was established based on echocardiographic and imaging findings. According to the modified Duke criteria, the patient met one major criterion (endocardial involvement with a large vegetation on the prosthetic mitral valve) and three minor criteria: fever >38 °C, a predisposing heart condition, and vascular phenomena (splenic, renal, mesenteric, and cerebral emboli). Inflammatory markers were markedly elevated (C-reactive protein 366 mg/L at admission) and progressively declined with empiric intravenous ceftriaxone and gentamicin.

Although her clinical course under antibiotics was favorable, the presence of a large, highly mobile vegetation associated with multiple systemic embolic events was considered by the endocarditis team to be a clear indication for urgent surgery. She was therefore transferred to a tertiary cardiac surgery center for reassessment and potential redo valve replacement. The patient ultimately underwent redo cardiac surgery; however, her postoperative course was unfavorable, and she unfortunately died shortly thereafter.

## Discussion

IE is a life-threatening disease [1]. Its pathogenesis is complex [5], resulting from infection of the heart valve and characterized by the formation of vegetations composed primarily of platelets, fibrin, and microorganisms [6]. Mechanical cardiac valves are valued for their efficacy and durability but carry an elevated thrombotic risk, necessitating lifelong anticoagulation. PVE is recognized as a more severe form of IE [2]. Two types of PVE are recognized: an early form, occurring within the first year after surgery, and a late form, developing more than one year postoperatively [7].

The incidence of IE is approximately three to 10 cases per 100,000 individuals, with mortality rates approaching 30% [7]. Its frequency has risen in recent years, including prosthetic valve IE [5], partly due to increasing intravenous drug use [8]. Other contributing factors include an aging population, greater use of prosthetic heart valves and cardiac implants, a growing number of intravascular procedures, and improved diagnostic capabilities [9]. The incidence of PVE is estimated at 1 to 2% per year, accounting for up to 20% of all IE cases. PVE is an uncommon but particularly severe complication of prosthetic cardiac valves [5], with mortality rates significantly higher than native valve IE, ranging from 22% to 40% [9].

Incomplete valvular seals are associated with increased thrombus formation, and the prosthetic valve itself may act as a nidus for infection [10]. Cardiac prostheses facilitate Staphylococcus aureus adherence via biofilm formation, promoting the development of PVE. Predictors of embolic events in IE include vegetation size >10 mm, high mobility, mitral valve or right-sided location, and elevated CRP [2].

Embolic events are severe complications of PVE, occurring in about 25% of patients at diagnosis. They result from migration of vegetation fragments to systemic or pulmonary circulation [11]. The brain is the most frequent site of embolization, followed by the spleen [1], kidneys, and lungs. Less common targets include the limbs and coronary arteries [2]. Reported incidence varies widely [1]. In the series by Menozzi et al., 61% of asymptomatic patients with left-sided IE had splenic infarctions [2]. Following stroke in PVE, about one-quarter of patients are not operated on due to the elevated hemorrhagic risk, with mortality in this group reaching nearly 65% [12]. In our case, imaging revealed multiple systemic emboli involving the spleen, right kidney, and superior mesenteric artery.

The clinical presentation of PVE generally mirrors that of native valve endocarditis, with nonspecific symptoms such as nausea, vomiting, and fever. Laboratory evaluation typically shows an inflammatory response with leukocytosis and elevated CRP. Staphylococcus aureus and Streptococcus species are the most frequent pathogens [13]. In our case, no microorganism was identified despite thorough microbiological assessment.

The cornerstone of treatment is pathogen-directed antimicrobial therapy, guided by susceptibility testing, with a typical duration of six weeks to reduce embolic risk. Surgical intervention to prevent embolic events is generally indicated in cases of persistent vegetations >10 mm or recurrent emboli despite appropriate antibiotic therapy [2], as recommended by the 2023 European Society of Cardiology guidelines (ESC). Stroke management in IE differs from that in the general population; according to the American Heart Association (AHA)/American Stroke Association, thrombolytic agents should be avoided due to the heightened risk of intracranial hemorrhage [1]. In prosthetic valve S. aureus IE with recent central nervous system embolism, anticoagulation may be withheld for two weeks and then cautiously reinitiated under close monitoring [14]. Both ESC and AHA guidelines advise delaying cardiac surgery for one month after ischemic stroke or intracranial hemorrhage [15], and recommend antibiotic prophylaxis before dental procedures, maintaining personal hygiene, and general precautions regarding body art (tattoos) [5].

This case highlights the need to consider infective endocarditis as a serious complication of prosthetic heart valves and to maintain a high index of suspicion for embolic events, which can be devastating and life-threatening. Early identification of embolic complications is crucial to guide timely and appropriate management and prevention strategies.

## Conclusions

Prosthetic valve endocarditis is a rare yet highly lethal complication of valve replacement, often beginning with subtle symptoms before rapidly progressing to severe events. In this case, the patient’s initial neurological deficit prompted comprehensive evaluation. Echocardiography revealed a large, mobile vegetation on the prosthetic mitral valve, a feature strongly linked to high embolic risk. CT imaging confirmed extensive embolization to multiple organs, highlighting the aggressive course of the disease. Early empiric intravenous antibiotic therapy resulted in clinical stabilization and a decline in inflammatory markers. Nevertheless, the combination of vegetation size, mobility, and recurrent embolic events constituted a clear indication for urgent surgical intervention. The patient subsequently underwent redo cardiac surgery; however, her postoperative course was unfavorable, and she unfortunately died shortly thereafter. This case emphasizes the importance of maintaining a high index of suspicion for embolic complications in prosthetic valve endocarditis. Timely diagnosis, multidisciplinary coordination, and adherence to guideline-based management are critical to improving patient outcomes.
